# Experimental Study of a Signal Modulation Method to Improve eLORAN Data Channel Communications

**DOI:** 10.3390/s20226504

**Published:** 2020-11-14

**Authors:** Chaozhong Yang, Yulin Wang, Shifeng Li, Wenhe Yan

**Affiliations:** 1 National Time Service Center, Chinese Academy of Sciences, Xi’an 710600, China; ycz@ntsc.ac.cn (C.Y.); wyulin@ntsc.ac.cn (Y.W.); ywh@ntsc.ac.cn (W.Y.); 2 Key Laboratory of Precise Positioning and Timing Technology, Chinese Academy of Sciences, Xi’an 710600, China

**Keywords:** signal modulation, eLORAN signal, LORAN data channel, data demodulation

## Abstract

There are mainly two types of data modulation methods used for enhanced LOng-RAnge Navigation (eLORAN) systems: pulse position modulation (PPM) and supernumerary interpulse modulation (SIM). The typical application for PPM is tri-state PPM (3S-PPM), also known as Eurofix. The typical application for SIM is ninth pulse modulation. Both of these methods are phase modulation methods. Phase modulation coding, a very mature technology, is used at present. To achieve a better demodulation success rate of eLORAN digital modulation signals at longer distances, a method of using the transmitting station duplex mode to transmit a digital modulation pulse group after LORAN-C transmitting a pulse group is proposed to realize modulation pulse on–off modulation. In this method, a broadcasting experiment was performed on the BPL (The call sign of eLORAN time service system in China) broadcaster station. After monitoring, a good receiving demodulation effect was initially obtained.

## 1. Introduction

The positioning, navigation, and timing (PNT) systems are important to the national economy and security of any country [1,2,3]. One of the high-precision ground-based PNT systems is Enhanced LOng-RAnge Navigation (eLORAN). It is the most widely used service among the long-standing and proven series of low-frequency LORAN systems [4,5,6].

The important difference between eLORAN and traditional LORAN-C is that eLORAN adds a data channel to the transmitted signal channel [7,8,9]. This data channel can convey correction information, warnings, and signal integrity information to the user’s receiver. The use of the data channel allows the eLORAN system to use nonprecision instruments to meet the very demanding requirements of landing aircraft, and to safely navigate the ship to the port under low visibility conditions. eLORAN can also provide differential data transmission and can be used as a global navigation satellite system (GNSS) redundant system [10,11,12].

eLORAN data communication has become an important direction in extending the application of eLORAN systems. There are mainly two types of data modulation methods used for eLORAN systems: tri-state pulse modulation and ninth pulse modulation. Tri-State Pulse Position Modulation (3S-PPM), also known as Eurofix, is standardized by Radio Technical Commission for Maritime services (RTCM) and International Telecommunication Union (ITU) [13,14,15,16]. Eurofix is adopted in China’s BPL long wave time service system and Changhe 2 navigation system. The signal-to-noise ratio (SNR) required for eLORAN data demodulation is higher than that for signal acquisition and detection. Both of Eurofix and ninth pulse modulation modes are phase modulation, which tends to cause phase blur and decrease the demodulation success rate when the propagation distance is far away [17,18,19,20].

According to China’s High Accuracy Ground-based Time Service System plan, the system plans to build three eLORAN time service broadcasting stations in western China, in order to adapt to the complex signal propagation environment in the inland areas of western China. In this paper, a modulation mode similar to On-Off Keying (OOK) is proposed. In additional pulse on-off modulation (APM), the success rate of demodulation is higher in the same environment and is more suitable for applications in complex propagation environments, and the performance of the APM modulation method is verified by modification of the BPL timing service system.

## 2. Materials and Methods

### 2.1. Tri-State Pulse Position Modulation (Eurofix)

Eurofix was first proposed by researchers at the Delft University of Technology. In the Eurofix system, the LORAN-C signal additional data channel does not affect the normal LORAN-C operation. The modulation method adopted by Eurofix is 3S-PPM, with a modulation amount of ±1 μs [21,22,23,24].

**Figure 1:**
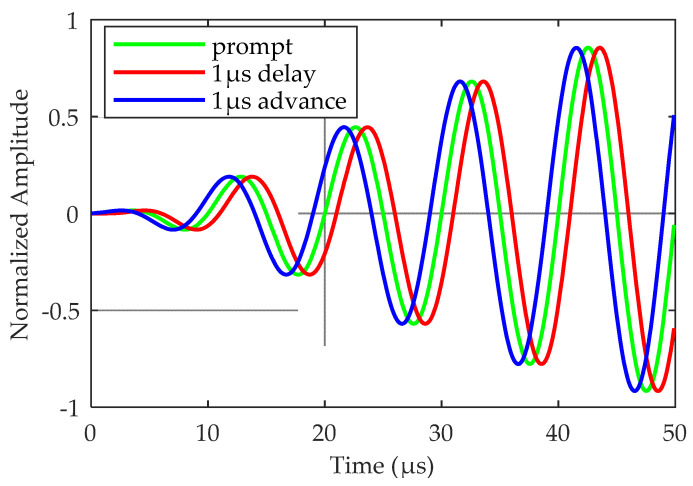
3S-PPM modulation.

3S-PPM modulates pulses 3–8 in each pulse group. The modulated signal consists of a 1 μs pulse transmission phase shift relative to the unmodulated pulse. The three possible states of modulation are given in Table 1 and Figure 1.

**Figure 2:**
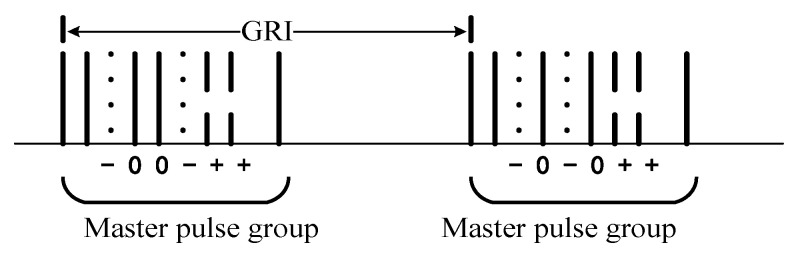
3S-PPM pattern.

In the 3S-PPM pulse group, the number of advanced and delayed pulses of a channel is equal. The modulation of six pulses in a pulse group will produce 141 possible modulation modes, of which 128 modes represent valid data, one mode represents no data transmission, and 12 models are not used. The 3S-PPM mode is shown in Figure 2. Each of the 128 effective modulation modes uniquely represents a 7-bit binary data block [13,14].

According to the different group repetition interval (GRI), the data rate is 70–175 bits/s, and uses forward error correction (Reed–Solomon encoding). The length of the Eurofix message is fixed at 210 bits and consists of 30 seven-bit words (GRI). Seventy bits are used to represent the application data, while the remaining 140 bits are used for forward error correction. The 70 bits of valid data include 4 bits for the message type, 52 bits for application data, and 14 bits for cyclic redundancy check (CRC) bits [25,26].

### 2.2. Ninth Pulse Modulation

**Figure 3:**
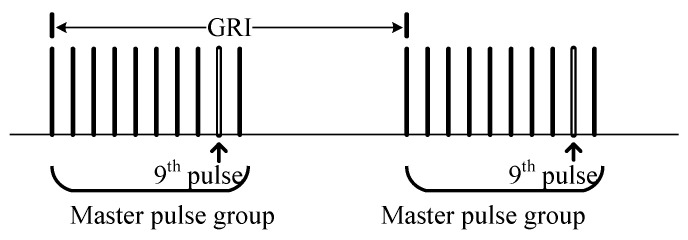
Ninth pulse pattern.

The ninth pulse technology for data transmission is the 32-ary PPM scheme. An additional pulse is inserted after the eighth pulse of the traditional LORAN pulse group. The 32 state PPM is used to represent the time delay from the zero symbol offset. Each signal position is defined by a 5-bit symbol, the first two digits indicate rough delayed, and the last three digits indicate fine delay. Figure 3 shows the ninth pulse pattern. In this way, the data transmission rate is 5 bits/GRI [15,27,28].

The zero sign offset of the additional pulse is 1000 µs. The delay time of the remaining 31 symbols relative to the zero symbol is microseconds of a specific time. The ideal delays are given by the formula:

(1) $$d_{i}=1.25mod\left(i,8\right)+50.625floor\left(i/8\right)$$

The actual delays are the ideal values shifted to coincide with the ticks of a 5-MHz clock. Table 2 lists the symbol *i* and corresponding time delay *d_i_* with respect to the zero symbol [15].

**Figure 4:**
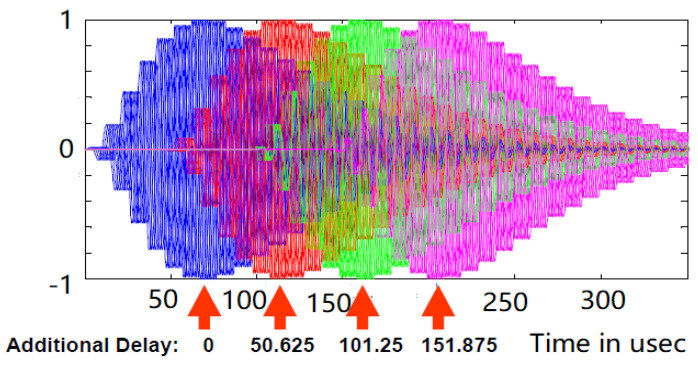
Ninth pulse modulation.

Figure 4 shows the ninth pulse modulation given in [27]. All modulation messages consist of three components: 4-bit for type, 41-bit for payload, and 75-bit for parity component. The message sending rate is 5 bits/GRI. The time length of the messages is 24 GRI (maximum of approximately 2.4 s).

### 2.3. Additional Pulse On–Off Modulation

**Figure 5:**
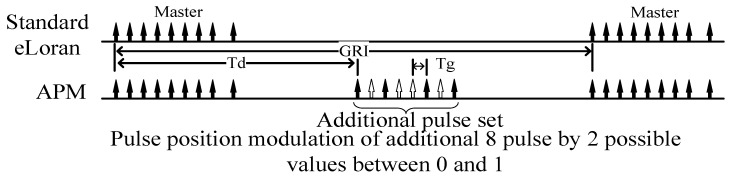
APM pattern.

In this article, an additional pulse on–off modulation method is proposed. Without changing the existing eLORAN system, eight pulses are added after the existing pulse set, and the presence or absence of pulses represents 1 and 0 in binary, similar to On–Off Key modulation. APM pattern is given in Figure 5.

As the pulse group is added after each main station, the GRI is the same, and there is no need to change the function of receiving signals. The pulse group takes the place of a navigation substation. Td is set and adjusted according to needs, the time delay is appropriately increased between the main station and the pulse group, the continuous working time of the transmitter is reduced, the cooling time of the transmitter is ensured, and the growth rate of the thermal load of the power equipment of the transmitting equipment is reduced.

APM pulse group starting position Td: In addition to the existing modulation signal 10 ms, there is a 50 ms gap for APM. However, considering the influence of sky waves, a 5 ms guard band is added before and after the existing system pulse, and at the same time, to avoid cross-interference from interfering with the existing pulse group and the APM pulse group at the same time, we considered selecting 31 ms as the APM pulse start time. Therefore, in this article, Td is set to 31 ms and Tg is 1 ms.

The first pulse of the eight additional pulses is the marking pulse, which is in the constant state (convenient for receiver detection); the second through eighth pulses of the additional pulses are in two states: presence or absence, corresponding to the binary data, with presence = “1” and absence = “0”; that is, each additional pulse set can represent 7-bit information.

Since each group of APM modulation corresponds to 7-bit binary code, and the Eurofix modulation of the main pulse group also corresponds to 7-bit binary code, the APM modulation uses the same frame structure and coding format.

One frame contains 30 GRI, the message information is 56 bits, the CRC (70, 56) cyclic redundancy code is 14 bits, and the RS (127, 107) error correction code is 140 bits, a total of 210 bits [29,30].

CRC is designed to be 14 bits, so the information code polynomial *M*(*x*) and generator polynomial *G*(*x*) are, respectively,

(2) $$M\left(x\right)=a_{55}x^{55}+a_{54}x^{54}+a_{53}x^{53}+\cdots +a_{1}x^{1}+a_{0}x^{0}$$

(3) $$G\left(x\right)=x^{14}+x^{13}+x^{7}+x^{5}+x^{4}+x$$

The RS code is designed to be 140 bits, so the primitive polynomial *P*(*x*) and the code generator polynomial *g*(*x*) are, respectively,

(4) $$P\left(x\right)=x^{7}+x^{3}+1$$

(5) $$g\left(x\right)=\left(x-a\right)\left(x-a^{2}\right)\left(x-a^{3}\right)\cdots \left(x-a^{20}\right)$$

## 3. Results

### 3.1. Signal Transmission Characteristics

The carrier frequency of the eLORAN signal is 100 kHz, and it is mainly transmitted through two paths to reach the receiving terminal: one is a ground wave signal that is transmitted along the surface of the earth to the receiving terminal; the other is a sky wave signal that is reflected by the ionosphere and reaches the receiving terminal. Among them, because the eLORAN ground wave signal propagates along the surface, the signal energy attenuates as the transmission distance increases, and the transmission delay increases accordingly. The speed of the signal attenuation and the transmission delay are mainly related to the transmission distance and path, the ground conductivity, relative permittivity, ground curvature, and atmospheric refractive index are related; the eLORAN sky wave signal is reflected and propagated through the ionosphere, and its arrival at the receiving terminal must lag behind the ground wave signal.

According to the “minimum performance standards for marine eLORAN receiving equipment” issued by Radio Technical Commission for Maritime Services (RTCM), the delay variation range of Loran-C’s sky-wave signal relative to its ground-wave signal is greater than 37.5 μs. The eLORAN receiver equipment will be capable of acquiring signals with field strength above 40 dBμV/m.

eLORAN ground wave signals are affected by the vertical component of ground wave field strength and ground wave propagation delay during transmission. Among them, the eLORAN ground wave field strength determines the range of the system, the sensitivity of the receiving terminal, and the design of the dynamic range, which is an important technical indicator of the eLORAN system; the propagation delay of the ground wave determines the path transmission between the eLORAN transmitting system and the receiver Delay accuracy.

eLORAN ground wave signals usually use the “Millington” semi-empirical method to calculate the signal field strength [20]:

(6) $$E\left(\mathrm{dB}\mu V/m\right)=109.54+101{\mathrm{lgP}}_{\Sigma }+20-20\mathrm{lg}d$$

$P_{\Sigma }$—radiation power of transmitting antenna, kW;
$W_{g}$—ground wave attenuation factor;
d—great circle distance between transmitting and receiving antennas, km;

The propagation delay of eLORAN ground wave signal can be expressed as:

(7) $$\begin{array}{cc} t_{g}\left(\omega \right) & =t_{0}+t_{w}\left(\omega \right)\\ & =\frac{d}{c}n_{s}\times 10^{6}+\left({\mathrm{argW}}_{g}/\omega \right)\times 10^{6} \end{array}$$

$t_{0}$—basic delay;
$t_{w}\left(\omega \right)$—secondary phase factor;
c—speed of light in free space, km/s;
d—the great circle distance between the transmitting and receiving antennas, km;
$n_{s}$—ground atmospheric refractive index;
$W_{g}$—ground wave attenuation factor;
ω—angular frequency, rad/s.

(8) $$W_{g}=F\left(q\right)+j\frac{1}{\mathrm{kd}}-{\left(\frac{1}{\mathrm{kd}}\right)}^{2}$$

(9) $$q=j{\left(\frac{Ka_{e}}{2}\right)}^{1/3}\frac{\sqrt{\varepsilon -1+j60\lambda \sigma }}{\varepsilon +j60\lambda \sigma }$$

K—wave number in the air, *K* = 2π/λ;
$a_{e}$—equivalent earth diameter, km;
ε—the relative dielectric constant of the ground;
σ—equivalent ground conductivity, S/m;
λ—the signal wavelength in vacuum, m.

### 3.2. Performance Comparison

Both the 3S-PPM and the ninth pulse modulations are types of phase modulation and have poor anti-noise performance. The eLORAN signal propagates through a long distance, which easily causes phase blur and makes phase recognition difficult. It also causes the demodulation success rate of the eLORAN receiver to decrease. APM modulation is similar to OOK modulation, which is easy to identify and conducive to receiver demodulation.

Assuming that the channel noise can be modeled by additive white Gaussian noise (AWGN), the probability of error can be computed as a function of the signal-to-noise ratio (SNR). Without considering the cross-interference, in this article, the probability of error of the aforementioned three modulation methods are analyzed and compared theoretically.

**Figure 6:**
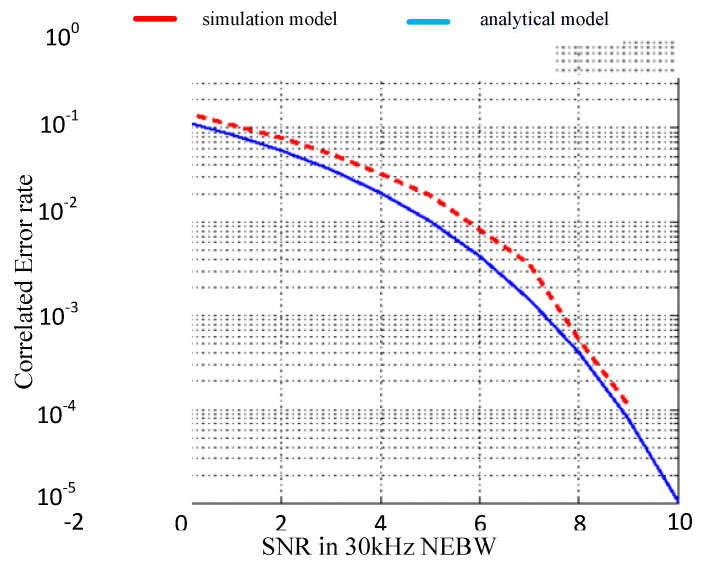
3S-PPM probability of error vs. SNR.

Figure 6 shows the error probability of 3S-PPM given in [26], and the difference between the analytical and simulation results is mostly due to the use of an ideal band-pass filter for the analytical model and a second-order Butterworth filter for the simulation. As shown in Figure 6, the red line represents the simulation model and the blue line the analytical model.

**Figure 7:**
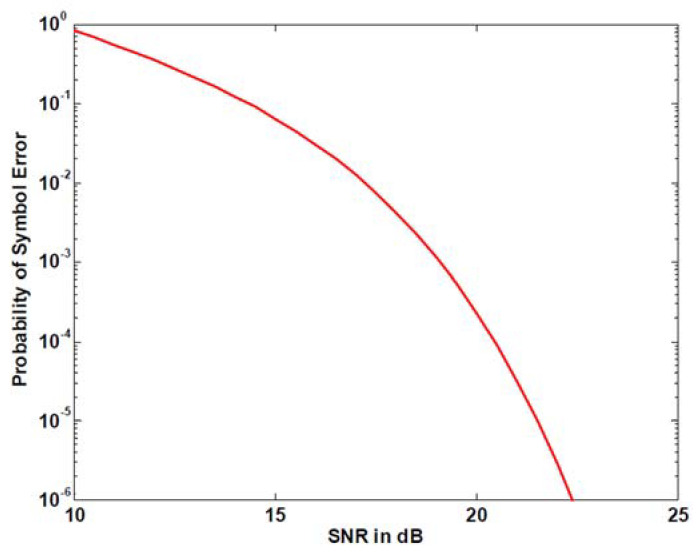
Ninth pulse probability of error vs. SNR.

Figure 7 shows the error probability of ninth pulse modulation given in [27].

**Figure 8:**
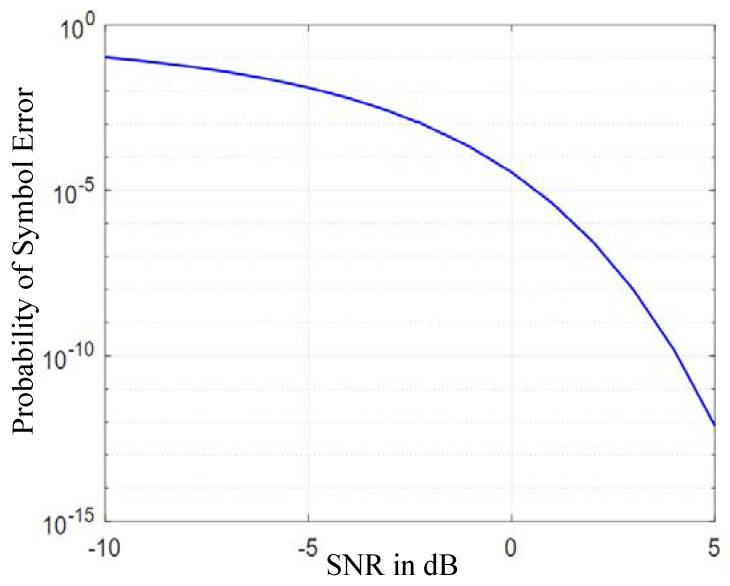
APM probability of error vs. SNR.

It can be seen from Figure 6, Figure 7 and Figure 8 that in the same SNR environment the bit error rate (BER) of ninth pulse modulation is the highest, while the BER of APM is the lowest.

This means that under the same working environment the range of APM signals demodulated by the receiver is larger than those of the 3S-PPM and ninth pulse signals.

### 3.3. Experimental Results

#### 3.3.1. Experiment Method

Experiments were performed in BPL timing service system. The BPL adopts the standard LORAN-C signal system with a GRI of 60 ms. The pulse group is the main station pulse (nine pulses). The pulse group digital modulation adopts the Eurofix modulation method to provide users with time information data. The transmitter uses a half-wave power synthesis solid-state transmitter and the timer has a duplex transmission function.

Transmitter device improvement: Setting the transmitter timer to duplex working mode, the signal format, and data modulation method of the main station pulse group and its equipment remain unchanged, the repetition period of the sub-station pulse group is adjusted to 60 ms, and the substation pulse group delay is adjusted to 31 ms. According to the Eurofix frame structure and coding format, a set of time information data is encoded to generate 7-bit binary code information to perform on–off control of the second- to eighth-bit pulses of the substation pulse group.

Improvement of receiving equipment: The original function of the receiver remains unchanged, and the modulation pulse waveform sampling, demodulation, and decoding functions need to be added only at 31 ms under normal reception conditions of the main station pulse signal.

**Figure 9:**
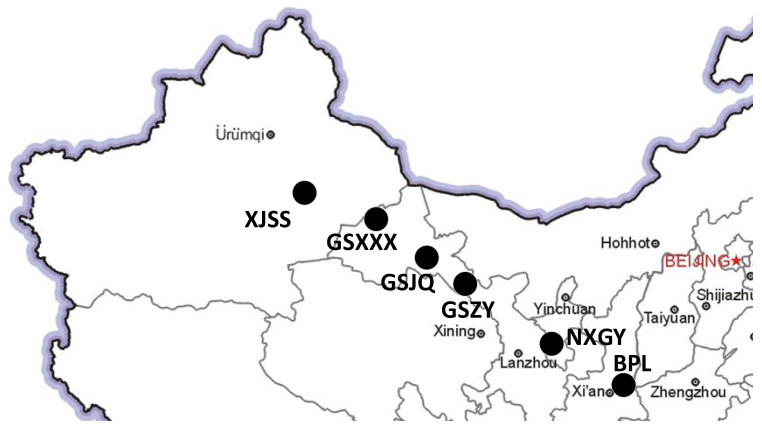
Test site distribution.

During the test, the content of the APM and 3S-PPM signal broadcasts was consistent, and the existing 3S-PPM receiver was modified to simultaneously demodulate APM signals to facilitate the demodulation success rate comparison between the two signal systems. The test site was chosen to be in the northwest of the BPL broadcast station to reduce cross-interference with the Changhe II system. The test sites are shown in Figure 9.

In this experiment, the existing BPL receiver was modified to have the ability to simultaneously demodulate 3S-PPM and APM signals. By analyzing the correctness of the time code information output by the BPL receiver, the decoding success rate of 3S-PPM and APM signal systems was compared.

By comparing the time difference between the 1PPS output by the BPL receiver and the 1PPS output by the GNSS receiver, the TOA error between the actual propagation delay value and the theoretical delay value is analyzed.

(10) $$TOA\;error=\bar{x}-\tau_{BPL}-\tau_{RCE}$$

$\bar{x}$—mean value of time difference measurement data;
$\tau_{BPL}$—the propagation delay of eLORAN ground wave signal;
$\tau_{RCE}$—BPL receiver delay value.

#### 3.3.2. Result Analysis

**Figure 10:**
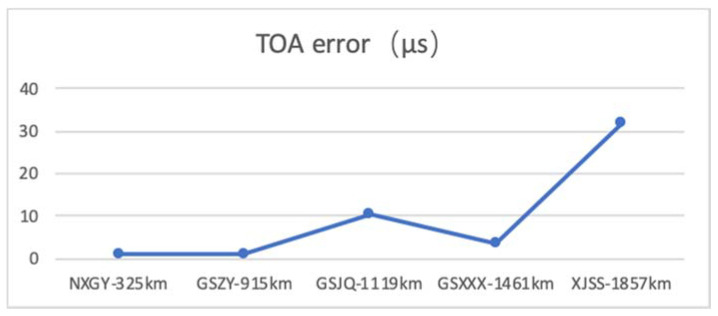
TOA error.

All test times were selected as day, because the night sky wave signal is enhanced, which will cause serious cross interference. The time of arrival (TOA) value of the eLORAN receiver is as shown in Figure 10. At all the test points, the TOA value is less than 37.5 μs, it can be determined that the receiver is locked to the ground wave signal.

**Figure 11:**
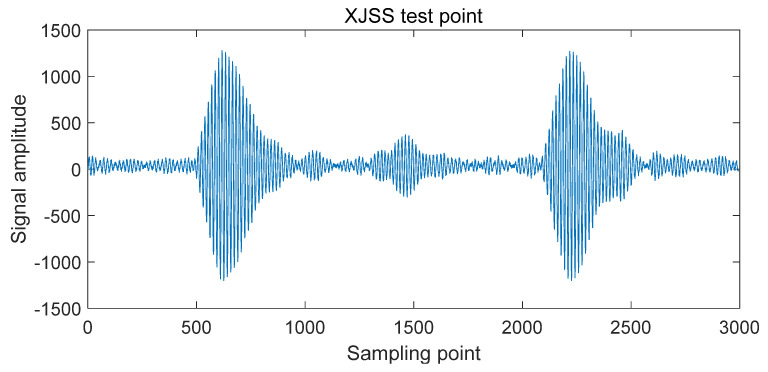
XJSS test point waveform.

At the XJSS test point, the TOA value has reached 31 μs, as shown in Figure 10, indicating that the sky wave signal has caused serious interference to the ground wave signal. The rising edge of the eLORAN pulse signal lags behind and the signal is distorted, as shown in Figure 11, causing deviations in standard zero-crossing detection. The performance of the standard zero crossing determines the error of the TOA value.

**Figure 12:**
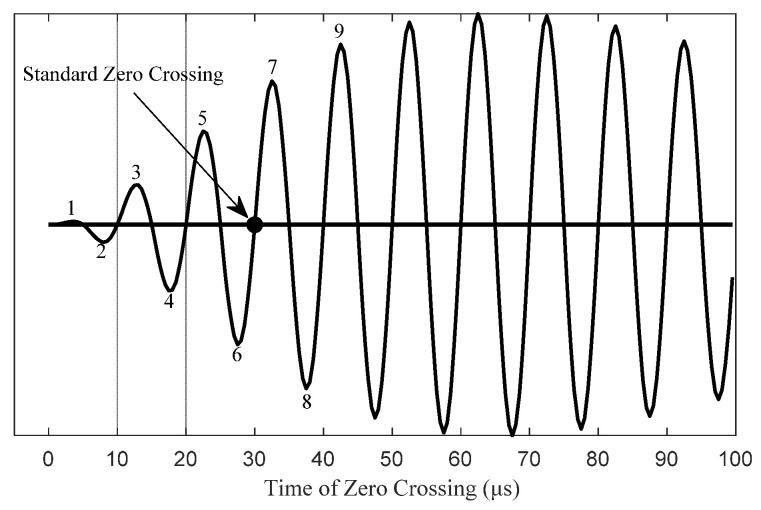
Standard zero crossing.

The standard zero crossing (SZC) is the positive zero crossing at the point 30 microseconds into a positively phase coded pulse on the antenna-current waveform, as shown in Figure 12. Given in [7], this zero crossing is phase-locked to the eLORAN station’s cesium time reference. The standard zero crossing is used as a timing reference for measurement of eLORAN signal specifications.

It can be seen from Table 3 that as the distance from the BPL station is increasingly further away, the decoding success rate of 3S-PPM and APM signals decreases. When field strength of the ground wave signal is weakened, such as the XJSS test point, the field strength has reached the minimum limit specified by RTCM, the demodulation success rate of 3S-PPM is greatly reduced, while the demodulation success rate of APM has maintained a high level. When the field strength of the test point becomes weaker, it is more susceptible to noise interference, which will cause difficulty in standard zero-crossing detection, and the error of TOA measurement results will increase. The demodulation success rate of 3S-PPM will decline rapidly, while the demodulation success rate of APM will still maintain a high level. However, at the same test point, the decoding success rate of APM signal is higher than that of 3S-PPM signal, which is consistent with the result of theoretical analysis.

## 4. Discussion

The realization and application of eLORAN system data channels technology can expand the application range of eLORAN system and build a relatively perfect PNT system. However, the existing demodulation method used in the eLORAN system cannot effectively suppress noise and interference. Therefore, with the development of modern eLORAN systems, a more advanced eLORAN signal modulation processing capability is required.

In order to adapt to the complex signal propagation environment in inland areas, increase the success rate of signal demodulation, and reduce the demodulation threshold of the receiver, this article proposed a new modulation method for eLORAN data channels, APM modulation, without changing the existing eLORAN system, eight pulses are added after the existing pulse set, and the presence or absence of pulses represents 1 and 0 in binary, similar to On–Off Key modulation. Therefore, the APM technology has a very low implementation complexity, and the performance of APM modulation with the prevailing 3S-PPM and ninth pulse modulations was compared. Theoretical analysis results show that the demodulation success rate of the APM signal modulation scheme is higher in the case of weak signals, that is, the signal field strength is small, and there is interference.

## 5. Conclusions

We set up an experimental verification platform that can broadcast an actual APM signal. The test results showed that when field strength of the ground wave signal is weakened, the field strength has reached the minimum limit specified by RTCM, the demodulation success rate of 3S-PPM is greatly reduced, while the demodulation success rate of APM has maintained a high level. Thus, APM modulation method has a higher engineering application value.

The APM modulation method is an extension of the compatibility of the original transmitting and receiving equipment. It does not affect the original signal format and modulation method, and doubles the amount of transmitted information.

In addition, it has certain advantages in resisting industrial interference and sky wave interference. The inadequacy of APM modulation lies in occupying the information resources of the transmission channel and increasing the load on the transmitter; the simultaneous use of multiple extended transmitting stations can easily cause cross-interference. In the planned follow-up work, we will carry out the cross-interference test of APM signals in the eastern part of China, this area is in the coverage of the Changhe 2 system.

## Figures and Tables

**Table 1:** States of modulation.

Pulse State	Transmission Time Minus Time of Reference Pulse (μs)	Indication
Advanced pulse	−1	−
Prompt pulse	0	0
Delayed pulse	+1	+

**Table 2:** Symbol delays from zero-symbol offset (μs).

i = [0, 7]		i = [8, 15]		i = [16, 23]		i = [24, 31]	
0	0.0	8	50.6	16	101.2	24	151.8
1	1.2	9	51.8	17	102.6	25	153.2
2	2.6	10	53.2	18	103.8	26	154.4
3	3.8	11	54.4	19	105.0	27	155.6
4	5.0	12	55.6	20	106.2	28	156.8
5	6.2	13	56.8	21	107.6	29	158.2
6	7.6	14	58.2	22	108.8	30	159.4
7	8.8	15	59.4	23	110.0	31	160.6

**Table 3:** Test data analysis.

Test Site	Distance from BPL (km)	Received Signal Estimated Field Strength (dBμv)	Decoding Success Rate	
			3S-PPM (%)	APM (%)
NXGY	325	81.9	98.5	100
GSZY	915	63.0	90.2	96.4
GSJQ	1119	58.0	83.9	96.7
GSXXX	1461	49.2	29.3	87.8
XJSS	1857	39.6	4.4	50.7
